# The guided understanding of implementation, development & education (GUIDE): a tool for implementation science instruction

**DOI:** 10.3389/frhs.2025.1654516

**Published:** 2025-09-26

**Authors:** Laura Ellen Ashcraft, Meghan B. Lane-Fall

**Affiliations:** ^1^ Division of Epidemiology, Department of Biostatistics, Epidemiology, and Informatics, Perelman School of Medicine, University of Pennsylvania, Philadelphia, PA, United States; ^2^ Leonard Davis Institute of Health Economics, University of Pennsylvania, Philadelphia, PA, United States; ^3^ Penn Implementation Science Center, University of Pennsylvania, Philadelphia, PA, United States; ^4^ Department of Anesthesiology, Vagelos College of Physicians and Surgeons, Columbia University in the City of New York, New York, NY, United States; ^5^ New York-Presbyterian/Columbia University Irving Medical Center, New York, NY, United States

**Keywords:** implementation science, education, implementation research logic model, pedagogy, implementation mapping

## Abstract

**Background:**

The use of implementation science in health research continues to increase, generating interest amongst those new to the field. However, conventional biomedical and health services research training does not necessarily equip scholars to incorporate theory-driven implementation science into their projects. Those new to IS may therefore struggle to apply abstract concepts from theory to their own work. In our teaching, we addressed this challenge by creating a practical teaching tool based on lessons from implementation mapping and the implementation research logic model (IRLM).

**The GUIDE:**

The tool is inspired by implementation mapping, the Implementation Research Logic Model, the ERIC implementation strategies, and Proctor's Outcomes Framework amongst other innumerable lessons from our experience as implementation scientists. We included sections to prompt learners to articulate the evidence-based practice of interest (including core and adaptable components) and the evidence-practice gap. The Guided Understanding of Implementation, Development & Education—GUIDE—and its corresponding prompts may provide a useful teaching tool to guide new users on incorporating implementation science into their evaluations. It also may help instructors illustrate how related implementation science concepts relate to each other over successive lessons or class sessions.

**Conclusion:**

This tool was developed from our experiences in teaching implementation science courses and consultation with new users in conjunction with common practices in the field including implementation mapping and the IRLM.

## Introduction

The use of implementation science (IS) continues to increase, creating a need for the development and deployment of practical teaching tools for widespread use. Ideally, such tools would be simple, a concept championed by Geoffrey Curran in his description of IS centered on “the thing” (1). The subsequent rapid adoption of this plain language terminology and explanation demonstrates the value of accessible language for learners and others who are new to the field. As instructors, we the authors educate and consult with trainees and investigators who want to integrate IS methods into their research. However, even scientists with robust training in biomedical and health services research struggle to apply IS to a given evidence-practice gap. Additionally, as implementation scientists, we partner with colleagues and scholars new to IS and must orient new staff to our projects. In such partnerships, we find ourselves teaching fundamental IS concepts to our teams to facilitate team-based inquiry.

In recognition of the difficulty we and others encounter in guiding new users to articulate “the thing” (including core and adaptable components) and the evidence-practice gap or problem, we developed and refined the Guided Understanding of Implementation, Development & Education (GUIDE) Tool. This tool combines and builds on implementation mapping (2) and the Implementation Research Logic Model (IRLM) (3), which are useful ways to systematically organize IS information and to guide the articulation of implementation strategies (using ERIC or Behaviour Change Techniques) and relevant outcomes (using the Proctor Outcomes Framework). The resulting GUIDE may help researchers and trainees new to IS to align inquiry with an IS lens and supports understanding of key IS aspects of IS.

The approach of taking an existing implementation tool designed for research and evaluation and adapting and simplifying it to support learners to IS was used before with Getting to Implementation (4) and Getting to Implementation-Teach (5). In the same way, we do not seek to strip any of the power and contributions of any individual existing implementation science resources and tools (e.g., implementation mapping, the IRLM, ERIC strategies, Proctor's outcomes, etc.). Instead, we seek to build on its foundation and popularity to introduce a new group of scientists to IS.

## The guided understanding of implementation, development & education (GUIDE) tool

**Figure 1:**
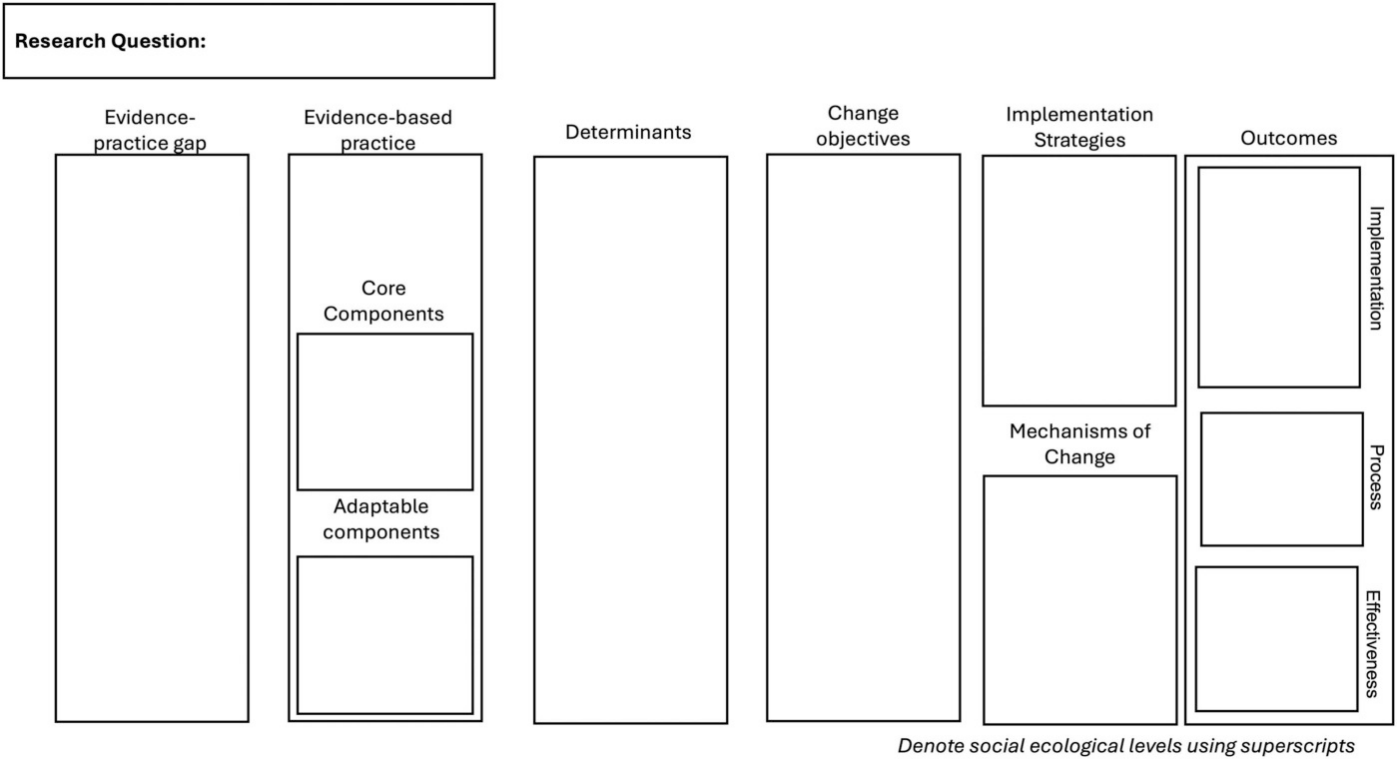
The guided understanding of implementation, development & education. The figure reads from left to right and can easily be reconfigured to read up-to-down or right-to-left to align with linguistic or cultural conventions.

The reasons trainees and investigators come to IS are heterogenous. We designed the GUIDE Tool to be responsive to these needs (see Figure 1). Supplementary Additional File 1 contains an editable version of the GUIDE Tool. Representative examples from our work include:

• A surgeon has an existing line of research and has been advised to add IS to their portfolio.
• A nurse practitioner has identified a practice of interest with limited evidence and seeks to identify the evidence-practice gap and test the effectiveness and implementation of said practice.
• A psychologist has an existing research question and study design and is considering finding a supplemental way to add IS approaches.

### GUIDE order of operations

Given this context, we include a research question box in the top left corner of the GUIDE Tool to allow users to anchor their learning and completion of the model within the framing of their existing research questions. There is not a preferred or linear flow to completing each of the sections of the GUIDE with learners' own knowledge or topic area***.*** Instead, each section represents a key aspect of implementation inquiry to be seriously considered by the user. Learners are introduced to each aspect of IS over time (see **Using the GUIDE to teach the key aspects of IS**). As knowledge increases, we encourage trainees to complete what portions they can with existing knowledge.

### When to use the GUIDE tool

We recommend the GUIDE Tool as an educational tool for teaching scholars and trainees new to IS about the key aspects of implementation research inquiry and evaluation over the course a semester or workshop session (see **Using the GUIDE Tool to teach the key aspects of IS** for more details). In conjunction with use as an educational tool, we have also found it helpful to use the GUIDE Tool to help scholars and trainees to organize existing information that they already to begin to think about designing an implementation study. In our experience, when novice IS users understand the key aspects of IS, they can begin to use IS evaluation tools as they were originally designed including, determinant frameworks [outlined by Nilsen (6)], change objectives (2), implementation strategies (7–9), mechanisms (10), and outcomes (6, 11, 12).

### Evidence-practice gap

The goal of the evidence-practice gap is to clearly identify the social problem of interest and the corresponding evidence-practice gap. Some questions to help articulate this gap include: What populations are impacted by this gap? In what places (geographic) or service settings does this gap exist? How do people experiencing this gap think about it?

### Evidence-based practice

We recognize that evidence-based practice, innovation, practice of interest or “the thing” have many synonyms and some synonyms have epistemological valences which go beyond the scope of this paper. That said, concretely identifying and specifying the “the thing” is critically important to implementation science. This includes identifying the level of evidence required for the specific practice to be considered “evidence-based.” We recognize that some fields require multiple large randomized controlled trials (e.g., pharmaceutical therapeutics) and in other fields innovations may be considered evidence-based anchored in less rigorous trial design due to ethical concerns (e.g., innovations targeting children or pregnant people).

Within the evidence-based practice, we prompt scholars to consider core and adaptable components. This practice helps to identify possible areas for scientific inquiry and recognizes that implementation in the real world requires compromise. We often recommend Figure 6 (p. 58)^1^ from the Implementation Facilitation Training Manual as an excellent resource for planning (13). We recognize that core and adaptable components of the evidence-based practice are often not specified in the existing literature. This introduces concerns not just because it can create complexity in implementing with fidelity, but also in that the core components of an EBP are often those associated with identifying the mechanisms of change.

#### Core components

The core components of an evidence-based practice are the defining characteristics of the innovation without which the innovation would not exist. A common question we ask is, “what has to happen in order for you to consider ‘the thing' to be ‘the thing’?”

#### Adaptable components

The adaptable components of an evidence-based practice are aspects that can be changed in small ways (e.g., in person or via telemedicine) or be skipped altogether. Scholars can offer tailoring and flexibility to implementers by prospectively identifying aspects of the innovation to be adapted (and save themselves headaches when “life” happens).

### Determinants

Determinants are factors that get in the way or support the ability of an individual, group, organization, or community to do “the thing.” Determinant frameworks are lists of potential determinants or constructs often organized into domains (6). Common determinant frameworks are the updated Consolidated Framework for Implementation Research (14) and the Tailored Implementation for Chronic Diseases (TICD) checklist (15). Historically, there has been a binary view of determinants in that they either barriers or facilitators. However, we take a valence agnostic approach in that over time and in evolving situations, determinants can act as both barriers and facilitators for the implementation of an innovation.

### Change objectives

Change objectives were first introduced by Fernandez and colleagues as part of implementation mapping (2). We view change objectives as the incremental steps between determinants and implementation strategies and provide transparency for why a given implementation strategy was selected and allude to potential mechanisms of change. The addition of change objectives to the original IRLM seeks to strengthen the important link between determinants and implementation strategies.

### Implementation strategies

Implementation strategies are “the stuff” we do to help people, places, groups, organizations, and communities do “the thing” (1). The most common taxonomy of 73 implementation strategies is the Expert Recommendations for Implementing Change (ERIC) and their nine clusters (7, 8). We find ERIC to be a helpful starting point for identifying potential implementation strategies and then encourage clear specification of the implementation strategy to promote clarity and transparency (16). Other compendia of implementation strategies, such as the Behaviour Change Techniques list from the Behaviour Change Wheel, could also be used here (9).

### Mechanisms of change

Mechanisms or mechanisms of change are the process through which an implementation strategy affects the targeted outcome (10). Mechanisms are a recent addition to the field of IS with diverse perspectives on their usefulness. Therefore, we encourage trainees to consider potential mechanisms of change, recognizing that implementation mechanisms are an emerging topic in the field.

### Outcomes

Outcomes include implementation outcomes in addition to service and patient outcomes (11, 17). Evaluation frameworks such as the Reach, Effectiveness, Adoption, Implementation and Maintenance framework (12, 18) and the Proctor framework (11) provide useful taxonomies for guiding the identification, specification, and measurement of implementation outcomes. We recognize the importance of implementation, process, and effectiveness outcomes and include distinct boxes within the Outcomes section for operationalizing each outcome of interest.

### Levels across the social ecological model

The social ecological model, first introduced by Urie Bronfenbrenner, identifies levels at which an activity may occur and recognizes the interaction across these levels (19) often visualized by concentric circles. The individual, microsystem, mesosystem, exosystem, and macrosystem can be tailored to an implementation context (e.g., patient, provider team, unit, hospital, health system, policy context). We have found that by identifying the social ecological model level across the GUIDE, especially for determinants, change objectives, and strategies, can allow for greater focus in evaluation. For example, when considering the embedded example of chronic pain management (see below), the change objective of “Demonstrate how to measure and diagnose chronic pain in primary care” occurs at both the individual and clinic level. Therefore, implementation strategies to accomplish this behavior should target individual clinicians and the clinic as a whole.

### Identifying gaps for evaluation or research design

We encourage users to print a copy of the GUIDE Tool or use Microsoft PowerPoint to complete the GUIDE with all the information they know from preliminary data collection, the peer-reviewed literature, and community knowledge. The completed GUIDE Tool may provide visual cues for existing gaps in knowledge and potential targets for future evaluation (3, 20) (see **Using the GUIDE to plan an IS study for novice IS users**). To further support these goals, we developed a complementary worksheet (see Supplementary Additional File 2) which includes prompts for each corresponding section of the GUIDE help those new to IS.

## Using the GUIDE tool to teach the key aspects of IS

We used the GUIDE as the organizing structure for the Foundations of Implementation Science course, the first introduction to IS as part of the IS Certificate program at the University of Pennsylvania. The goal of this course is to introduce trainees and scholars new to implementation science to the key aspects of the field and provide examples of how they can apply IS principles to their own scholarship.

**Figure 2:**
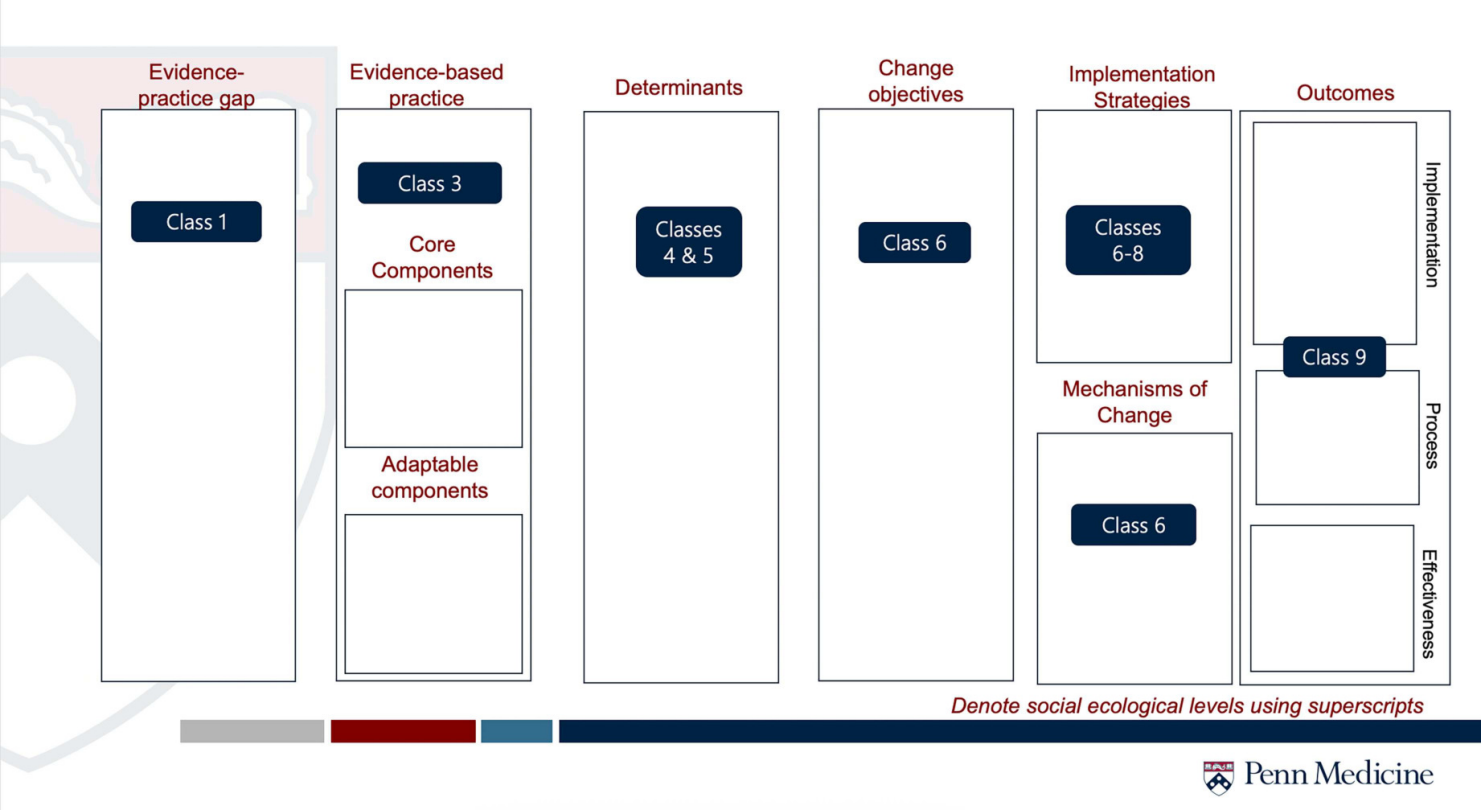
Example of GUIDE for teaching. This is a slide used throughout the semester of Foundations of Implementation Science course to help orient the learner to how the current session fits into the larger picture of IS.

The original course was developed and taught by the senior author (MLF) in Fall 2023. In Fall 2024, the authors (LEA and MLF) co-taught the course and aligned the content with each aspect of the GUIDE Tool (see Figure 2). The first author (LEA) taught a workshop for Doctor of Social Work students, several guest lectures, and is again teaching the Foundations course in Fall 2025 using the GUIDE in an organizing capacity and teaching tool. We further operationalize how each component of IS represented in the GUIDE fits into learning through course objectives listed in Table 1.

**Table 1:** Corresponding learning objectives for the GUIDE.

Class	Learning objectives
Evidence-practice gap (Class 1)	• Present a high-level overview of implementation science • Discuss competencies in implementation science
Evidence-based practice, Core & Adaptable components (Class 3)	• Explain commonly used standards of evidence in research that lead to implementation • Describe reasons aside from evidence that there might be tension for change • Discuss challenges in defining “the thing” • Explain the concept of intervention adaptation and why it matters to implementation • Describe the core components of an evidence-based practice (Class 5)
Determinants (Classes 4 & 5)	• Explain the relationship between the Behavior Change Wheel and the COM-B model • List the five CFIR domains • Apply a TMF to an evidence-practice gap of interest
Change Objectives (Class 6)	• Define change objectives • List 2–3 approaches to articulating change objectives
Implementation strategies (Classes 6–8)	• Explain how Proctor's model relates implementation strategies to outcomes • Define implementation strategies • Review different types and classifications of implementation strategies • Discuss how to select and report implementation strategies • Describe the role of organizations in EBP implementation • Discuss the role of policy in fostering change in health and social services • Explain the power and perils of using policy to address implementation challenges
Mechanisms of change (Class 6)	• Explain the concept of mechanisms of change
Outcomes (Class 9)	• Explain how implementation outcomes relate to effectiveness outcomes • Define the initial eight implementation outcomes from the Proctor outcomes framework • Discuss the merits of qualitative, quantitative, and mixed methods measurement of implementation outcomes • Explain how implementation outcomes relate to effectiveness outcomes • Define the initial eight implementation outcomes from the Proctor outcomes framework • Discuss the merits of qualitative, quantitative, and mixed methods measurement of implementation outcomes

Listed Learning Objectives are only those relevant to the GUIDE Tool. Other topics covered but not listed include, but not limited to: overview of theories, models, and frameworks, theoretical underpinnings of the field, and dissemination.

Throughout the course, the GUIDE functions as a roadmap for learners to identify how what they are learning fits within the greater picture of IS. For example, in Classes 6, 7, and 8 we discuss implementation and dissemination strategies. By introducing the concept later in the semester, we work to support learners' understand the importance of how implementation strategies connect to both change objectives and implementation determinants. Using the GUIDE, learners may visualize how these strategies connect to mechanisms and implementation outcomes.

Not all class sessions are represented by the GUIDE as a typical semester has 12–15 weeks. This allows us to be responsive to the needs of our learners and incorporate other important topics in implementation science such as implementation mapping (Class 10), community engagement in non-health fields (Class 11), best practices in reporting IS studies (Class 13); and a “Choose your own adventure” session where we discuss IS topics of interest that arise throughout the semester (Class 14).

## Using the GUIDE to plan an IS study for novice IS users

As previously mentioned, some IS trainees come to the field with existing research or a study in mind without designing specifically for IS. Here, we introduce a case example of how the GUIDE can be applied based on a previous qualitative study conducted by the first author (LEA) while they were a PhD-student and a novice IS user. The study examined the determinants of dissemination and implementation of evidence-based chronic pain management in primary care (21). Briefly, interviews were conducted with primary care providers across multiple health systems to better understand factors that impact their ability to learn about (dissemination) and use (implementation) evidence-based chronic pain management, primarily focusing on non-pharmacologic approaches.

**Figure 3:**
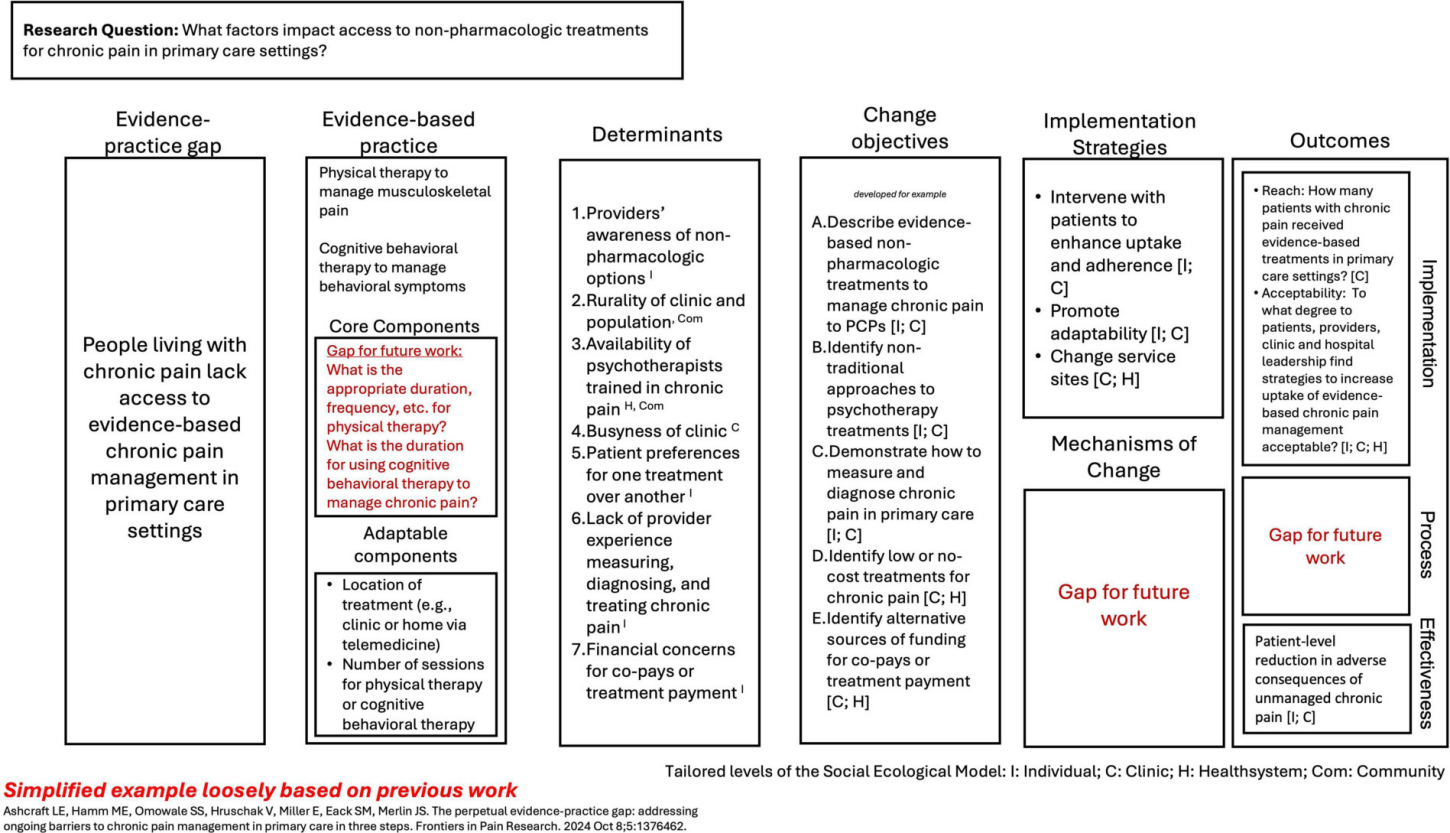
Example of GUIDE use in chronic pain management. The Figure provides an example of how the GUIDE can be applied to an existing research project for a scholar new to implementation science. Each section was completed using information from a qualitative research study in response to the prompts. For this example, the social ecological model was delineated as the individual, clinic, health system, and community. We aligned both determinants and change objectives with the social ecological model to help guide future data collection and analysis.

As shown in Figure 3 (below), we used the study results to complete the GUIDE and identified gaps for future work. For example, starting with EBP core components, we recognized that while PCPs gave some examples of evidence-based chronic pain management, they did not fully articulate (or did not know) what duration, dosage, or specific aspects are required for physical therapy and/or cognitive behavioral therapy to meet the needs of people living with chronic pain. This highlights a larger gap in the field of innovation development that impacts implementation science. Additionally, the interviews did not discuss potential mechanisms of change to support a potential causal pathway. Hypothetically, if there existed a body of literature which has already explored mechanisms of change in this setting, we could add this to the GUIDE.

We identified at what level of the social ecological model each of the determinants and change objective would act upon. For example, the determinant of the degree to which clinic rurality and the clinic population composition impacts the ability to implement evidence-based chronic pain management is a community-level factor. In contrast, the change objective to identify alternative sources of funding for co-pays or treatment payment occurs both at the clinic level and at the health system level (depending on the organizational structure).

This example shows how the GUIDE may help to organize existing knowledge about an evidence-practice gap into the language of IS and can identify key gaps in knowledge for future inquiry.

## Discussion

Implementation science seeks to close the evidence-practice gap between what is currently happening and what we want to happen in an ideal world. We developed the IRLM using the existing knowledge base from implementation mapping, the IRLM, and other resources including ERIC and the Proctor outcomes framework to develop a teaching tool for learners to IS. We hold that the innumerable lessons and evidence already learned in the field should be accessible to both scholars who spend most of their professional lives thinking about implementation science and to community providers who are looking to address a problem they see.

## Data Availability

The original contributions presented in the study are included in the article/Supplementary Material, further inquiries can be directed to the corresponding author.

## Footnotes

^1^ https://www.queri.research.va.gov/tools/Facilitation-Manual.pdf

## References

[1] Curran GM. Implementation science made too simple: a teaching tool. Implement Sci Commun. (2020) 1:1–3. 10.1186/s43058-020-00001-z 32885186 PMC7427844

[2] Fernandez ME ten Hoor GA van Lieshout S Rodriguez SA Beidas RS Parcel G Implementation mapping: using intervention mapping to develop implementation strategies. Front Public Health. (2019) 7:158. 10.3389/fpubh.2019.00158 31275915 PMC6592155

[3] Smith JD Li DH Rafferty MR. The implementation research logic model: a method for planning, executing, reporting, and synthesizing implementation projects. Implement Sci. (2020) 15:84. 10.1186/s13012-020-01041-8 32988389 PMC7523057

[4] Yakovchenko V Rogal SS Goodrich DE Lamorte C Neely B Merante M Getting to implementation: adaptation of an implementation playbook. Front Public Health. (2023) 6:10. 10.3389/fpubh.2022.980958 PMC9853037 36684876

[5] Rogal SS Jonassaint C Ashcraft L Freburger J Yakovchenko V Kislovskiy Y Getting to implementation (GTI)-teach: a seven-step approach for teaching the fundamentals of implementation science. J Clin Transl Sci. (2022) 6:e100. 10.1017/cts.2022.420 36106128 PMC9428668

[6] Nilsen P. Making sense of implementation theories, models and frameworks. Implement Sci. (2015) 10:53. 10.1186/s13012-015-0242-0 25895742 PMC4406164

[7] Powell BJ Waltz TJ Chinman MJ Damschroder LJ Smith JL Matthieu MM A refined compilation of implementation strategies: results from the expert recommendations for implementing change (ERIC) project. Implement Sci. (2015) 10:21. 10.1186/s13012-015-0209-1 25889199 PMC4328074

[8] Waltz TJ Powell BJ Matthieu MM Damschroder LJ Chinman MJ Smith JL Use of concept mapping to characterize relationships among implementation strategies and assess their feasibility and importance: results from the expert recommendations for implementing change (ERIC) study. Implement Sci. (2015) 10:109. 10.1186/s13012-015-0295-0 26249843 PMC4527340

[9] Michie S Atkins L West R. The Behaviour Change Wheel. A Guide to Designing Interventions, 1st ed. Great Britain: Silverback Publishing (2014), Vol. 1003, p. 1010.

[10] Lewis CC Klasnja P Powell BJ Lyon AR Tuzzio L Jones S From classification to causality: advancing understanding of mechanisms of change in implementation science. Front Public Health. (2018) 6:136. 10.3389/fpubh.2018.00136 29868544 PMC5949843

[11] Proctor E Silmere H Raghavan R Hovmand P Aarons G Bunger A Outcomes for implementation research: conceptual distinctions, measurement challenges, and research agenda. Adm Policy Ment Health. (2011) 38:65–76. 10.1007/s10488-010-0319-7 20957426 PMC3068522

[12] Glasgow RE Vogt TM Boles SM. Evaluating the public health impact of health promotion interventions: the RE-AIM framework. Am J Public Health. (1999) 89:1322–7. 10.2105/AJPH.89.9.1322 10474547 PMC1508772

[13] Ritchie M Dollar K Miller C Smith J Oliver K Bim B Using Implementation Facilitation to Improve Healthcare (Version 3). Veterans Health Administration, Behavioral Health Quality Enhancement Research Initiative (QUERI) (2020). Available online at: https://www.queri.research.va.gov/tools/Facilitation-Manual.pdf

[14] Damschroder LJ Reardon CM Widerquist MAO Lowery J. The updated consolidated framework for implementation research based on user feedback. Implement Sci. (2022) 17:1–16. 10.1186/s13012-022-01245-0 36309746 PMC9617234

[15] Flottorp SA Oxman AD Krause J Musila NR Wensing M Godycki-Cwirko M A checklist for identifying determinants of practice: a systematic review and synthesis of frameworks and taxonomies of factors that prevent or enable improvements in healthcare professional practice. Implement Sci. (2013) 8:1–11. 10.1186/1748-5908-8-35 23522377 PMC3617095

[16] Proctor EK Powell BJ McMillen JC. Implementation strategies: recommendations for specifying and reporting. Implement Sci. (2013) 8:139. 10.1186/1748-5908-8-139 24289295 PMC3882890

[17] Proctor EK Landsverk J Aarons G Chambers D Glisson C Mittman B. Implementation research in mental health services: an emerging science with conceptual, methodological, and training challenges. Adm Policy Ment Health. (2009) 36:24–34. 10.1007/s10488-008-0197-4 19104929 PMC3808121

[18] Feldstein AC Glasgow RE. A practical, robust implementation and sustainability model (PRISM) for integrating research findings into practice. Jt Comm J Qual Patient Saf. (2008) 34:228–43. 10.1016/S1553-7250(08)34030-6 18468362

[19] Bronfenbrenner U. Ecological systems theory (1992). In: Bronfenbrenner, editor. Making Human Beings Human: Bioecological Perspectives on Human Development. Thousand Oaks, CA: Sage Publications Ltd (2005). p. 106–73.

[20] Merle JL Sloss EA Sanuade OA Lengnick-Hall R Meza R Golden C Refining the implementation research logic model: a citation analysis, user survey, and scoping review protocol. Front Health Serv. (2024) 4. 10.3389/frhs.2024 39512272 PMC11540644

[21] Ashcraft LE Hamm ME Omowale SS Hruschak V Miller E Eack SM The perpetual evidence-practice gap: addressing ongoing barriers to chronic pain management in primary care in three steps. Front Pain Res. (2024) 5. 10.3389/fpain.2024.1376462 PMC11493740 39439739

