# Correction: The guided understanding of implementation, development & education (GUIDE): a tool for implementation science instruction

**DOI:** 10.3389/frhs.2025.1724907

**Published:** 2025-10-29

**Authors:** Frontiers Production Office

**Affiliations:** Frontiers Media SA, Lausanne, Switzerland

**Keywords:** implementation science, education, implementation research logic model, pedagogy, implementation mapping

A Correction on The guided understanding of implementation, development & education (GUIDE): a tool for implementation science instruction Ashcraft LE and Lane-Fall MB (2025). Front. Health Serv. 5:1654516. doi: 10.3389/frhs.2025.1654516

The incorrect file for Table 1 in the Supplementary Material was published with the original version of this paper. The supplementary file Table 1 has now been replaced with the correct version.

The original version of this article has been updated.

