# Supplementary material for: The guided understanding of implementation, development & education (GUIDE): a tool for implementation science instruction
Source: Front Health Serv. 2025 Sep 26;5:1654516. doi: 10.3389/frhs.2025.1654516 (PMC12510919; doi:10.3389/frhs.2025.1654516)
Supplement: Supplementary file 1 [file Presentation1.pptx]

## Slide 1
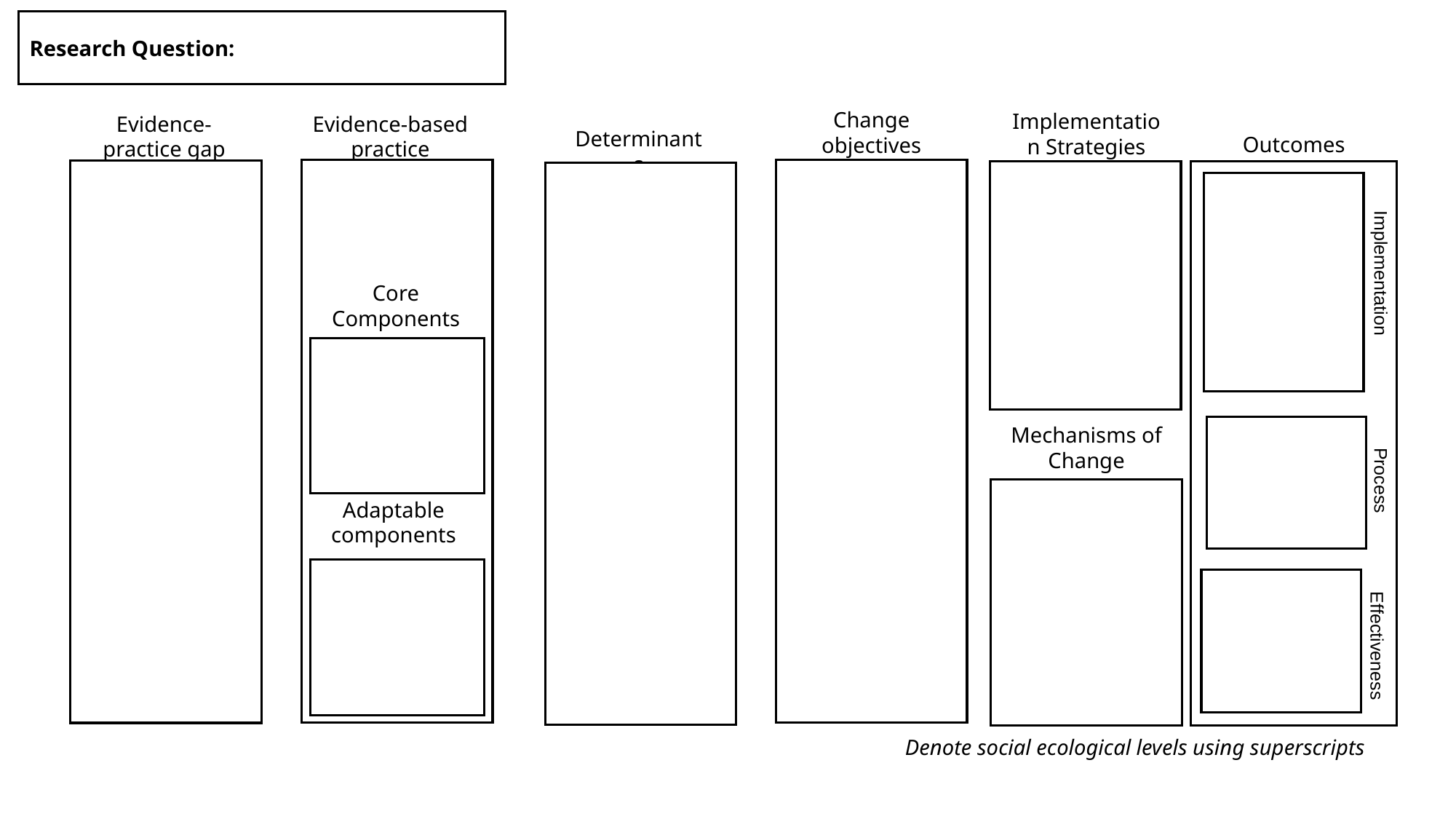

Research Question:
Change objectives
Implementation Strategies
Evidence-practice gap
Evidence-based practice
Determinants
Outcomes
Core Components
Adaptable components
Implementation
Mechanisms of Change
Process
Effectiveness
Denote social ecological levels using superscripts
