# Supplementary material for: The guided understanding of implementation, development & education (GUIDE): a tool for implementation science instruction
Source: Front Health Serv. 2025 Sep 26;5:1654516. doi: 10.3389/frhs.2025.1654516 (PMC12510919; doi:10.3389/frhs.2025.1654516)
Supplement: Supplementary file 2 [file Table1.docx]

Implementation Science Research Worksheet

*Developed by LauraEllen Ashcraft, PhD, MSW*

**Evidence-practice gap**

***Part 1: Is this an appropriate use of implementation science?***

**The problem**

1. What is the evidence-practice gap?
2. Who experiences this evidence-practice gap?
3. Where does this evidence-practice gap exist?
4. What do people experiencing the evidence-practice gap think about the gap?
5. How is this problem typically discussed?

**Evidence-based practice**

**The “thing”**

1. What is the evidence-based practice (EBP)?
2. What is the evidence supporting the EBP?
3. What is the evidence for this specific population?
4. What is the evidence for this specific setting?
5. What parts of the EBP are needed?

*Caveat 1: If there is some knowledge*^[[1]](#footnote-1)^ *supporting the EBP either in the same population or setting—it’s time for an implementation study!*

*Caveat 2: if you do not yet have a solution or EBP, you likely are not ready to use implementation science. That said, there are ways to incorporate implementation science in intervention/EBP development.*

***Part 2: Beginning to use implementation science, what do you already know?***

**Determinants**

**Identify determinants**

1. What are the strengths in the setting related to the problem?
2. What are the primary challenges to addressing the problem?
3. What do people think about the EBP?

*Resources:* The Consolidated Framework for Implementation Research by Damschroder and colleagues (2022) is a taxonomy of implementation determinants.

**Change Objectives**

**Change objectives**

1. What specific behaviors that need to happen to reach identified goals?

**Implementation Strategies**

**Implementation strategies**

1. Based on existing knowledge, what is the best way to put the EBP into practice?
2. What are strategies have others used to put the EBP into practice?

*Resources:* The Expert Recommendations for Implementing Change or ERIC is a taxonomy of implementation strategies; Proctor and colleagues (2013) provide guidance in how to operationalize implementation strategies.

**Mechanisms of Change**

**Mechanisms of change**

1. What is the process or event by which an implementation strategy(ies) achieves its intended affect?

*Resources:* Lewis and colleagues (2018) provide a useful description of the need for and examples of mechanisms of change.

**Outcomes**

**Outcomes**

1. How well does the EBP get to the people it’s designed to support?
2. How well does the EBP improve clinical or process outcomes?
3. How well is the EBP adopted by providers or clinicians who are intended to use?
4. How well is the EBP being utilized as intended?
5. How well is the EBP being sustained over time?
6. Regarding the process of implementing the EBP, how much and how well is utilization going?
7. Are these outcomes perceptual or behavioral?
8. What are process outcomes related to the implementation of the EBP?
9. What are effectiveness outcomes of interest?

*Resources:* The Reach, Effectiveness, Adoption, Implementation, and Maintenance (RE-AIM) framework by Glasgow and colleagues (1999, 2008) is a useful tool for helping to define implementation outcomes; Proctor and colleagues (2011) provide a list of and definitions for implementation outcomes.

**Levels across the Social Ecological Model**

**Levels of Change (consider for each of the previous sections within Part 2)**

1. What changes(s) needs to happen at the provider/clinician level?
2. What changes(s) needs to happen at the unit or group level?
3. What changes(s) needs to happen at the organization level?
4. What changes(s) needs to happen at the community level?
5. What changes(s) needs to happen at the policy/societal level?

***Part 3: Designing your implementation science evaluation***

**Identifying gaps for evaluation or research design**

**Developing an implementation research question**

1. What is the implementation gap of most need based on what you already know?
2. What are the priorities of the community?
3. What is feasible?

**Incorporating implementation science theories, models, and frameworks**

1. What has been used before in this 1) social problem, 2) evidence-based practice, and/or 3) setting?
2. Do you need a determinant, process, or outcome framework?
3. How can you incorporate factors of equity and justice?

*Resources: Nilsen (2015) provides an excellent summary of theories, models, and frameworks in implementation science.*

**Selecting your implementation study type**

1. Based on the existing level of knowledge, what type of implementation study is most appliable

*Resources:* The implementation subway line by Lane-Fall and colleagues (2019) provides useful insights into what type of implementation study is most relevant.

**Study design**

1. What methodological approach is most appropriate given your topic, setting, and available resources?
2. Will you use prospective or retrospective data collection?
3. Will analysis be qualitative, quantitative, or mixed methods?
4. Will you conduct hypothesis testing?
5. How will your implementation be sustainable beyond the study timeframe?
6. How might your EBP, strategies, or context need to be adapted?
7. How will you engage community/clinical partners throughout the design and study implementation process?

*Resources:* FRAME (Wiltsey-Stirman et al., 2019) and FRAME-IS (Miller et al., 2021) are useful ways to help think about and track adaptations throughout the implementation process.

*Caveat: There are often no “right” answers to these questions. Even in the same circumstances, different study designs based on these prompts are equally justifiable.*

1. Justice and equity-oriented implementation acknowledges that knowledge takes many forms. This may include empirical knowledge, community knowledge, lived experience, etc. [↑](#footnote-ref-1)
